# Bayesian Spatiotemporal Pattern and Eco-climatological Drivers of Striped Skunk Rabies in the North Central Plains

**DOI:** 10.1371/journal.pntd.0004632

**Published:** 2016-04-29

**Authors:** Ram K. Raghavan, Cathleen A. Hanlon, Douglas G. Goodin, Rolan Davis, Michael Moore, Susan Moore, Gary A. Anderson

**Affiliations:** 1 Kansas State Veterinary Diagnostic Laboratory and Department of Diagnostic Medicine/Pathobiology, College of Veterinary Medicine, Kansas State University, Manhattan, Kansas, United States of America; 2 Centers for Disease Control and Prevention, Atlanta, Georgia, United States of America; 3 Department of Geography, College of Arts and Sciences, Kansas State University, Manhattan, Kansas, United States of America; The Wistar Institute, UNITED STATES

## Abstract

Striped skunks are one of the most important terrestrial reservoirs of rabies virus in North America, and yet the prevalence of rabies among this host is only passively monitored and the disease among this host remains largely unmanaged. Oral vaccination campaigns have not efficiently targeted striped skunks, while periodic spillovers of striped skunk variant viruses to other animals, including some domestic animals, are routinely recorded. In this study we evaluated the spatial and spatio-temporal patterns of infection status among striped skunk cases submitted for rabies testing in the North Central Plains of US in a Bayesian hierarchical framework, and also evaluated potential eco-climatological drivers of such patterns. Two Bayesian hierarchical models were fitted to point-referenced striped skunk rabies cases [n = 656 (negative), and n = 310 (positive)] received at a leading rabies diagnostic facility between the years 2007–2013. The first model included only spatial and temporal terms and a second covariate model included additional covariates representing eco-climatic conditions within a 4km^2^ home-range area for striped skunks. The better performing covariate model indicated the presence of significant spatial and temporal trends in the dataset and identified higher amounts of land covered by low-intensity developed areas [Odds ratio (OR) = 3.41; 95% Bayesian Credible Intervals (CrI) = 2.08, 3.85], higher level of patch fragmentation (OR = 1.70; 95% CrI = 1.25, 2.89), and diurnal temperature range (OR = 0.54; 95% CrI = 0.27, 0.91) to be important drivers of striped skunk rabies incidence in the study area. Model validation statistics indicated satisfactory performance for both models; however, the covariate model fared better. The findings of this study are important in the context of rabies management among striped skunks in North America, and the relevance of physical and climatological factors as risk factors for skunk to human rabies transmission and the space-time patterns of striped skunk rabies are discussed.

## Author Summary

Despite the long recognition that skunks are an important reservoir host for rabies, the control of this disease among this host has not been achieved, and the disease is currently only passively monitored in North America. The need for rabies control among striped skunks is, however, well acknowledged, and reports of occasional spill-over of skunk variant rabies viruses to non-reservoir species, including some domestic animals, remains a cause for public health concern and a major roadblock for eradicating rabies from North America. An understanding of the spatial and temporal dynamics of diseases is important in management and for setting future research agendas, and such knowledge could assist in effective striped skunk rabies control. In this study, we evaluated whether rabies among striped skunk cases submitted for testing in the North Central Plains exhibit discernable spatial and temporal patterns, and if there are any eco-climatic factors that influenced such patterns. Our findings indicate that the year-to-year and spatial origins of rabies incidences in the states of Kansas and Nebraska in the North Central Plains are currently stable, and certain physical environment (developed low-intensity areas and patch fragmentation) and climatic (diurnal temperature range) factors play an important role in determining such temporal and spatial patterns.

## Introduction

Rabies is one of the oldest known zoonosis, a central nervous system disease of mammals caused by viruses in the Rhabdoviridae family. Rabies continues to kill people throughout the world although human deaths due to this disease in the US have become increasingly rare [1]. The main global source of rabies occurs among domestic dogs but dog-to-dog transmitted rabies has been pushed to near extinction in North America since the mid-20^th^ century due to intensive dog vaccination campaigns and stray dog control [1]. Numerous variants of the virus however circulate in wildlife today in North America, primarily among those species in the orders Carnivora and Chiroptera. In California, Texas and the north-central United States, the disease is well established among skunks in the *Mephitis* genera, and although all skunk species are susceptible, the striped skunk, *Mephitis mephitis* is the most reported rabid species to diagnostic facilities in these regions [2] [3]. Two distinct variants of the virus are recognized for striped skunk rabies in the central Midwestern US, one occurring in the South Central and the other in North Central Plains [2].

Striped skunks are widely distributed habitat-generalists in North America, with an ability to colonize periurban and rural environments [4]. Efforts to control or manage rabies among skunks has been a challenge even though they have been known as a major terrestrial reservoir for rabies in central and western US and as well as a public health concern for many decades [5–7]. Unlike the widely acknowledged success of oral rabies vaccine (ORV) programs in immunizing raccoons, foxes and coyotes [8–10], ORV baits are less effective on striped skunks and has not produced detectable levels of immunity [10] [11]. The authors are aware of at least one current effort in the Midwestern US that aims to understand the efficacy of ORV baits in immunizing striped skunks in some parts of Texas but results have not been brought to public attention at the time of this study. It can be safely said therefore that striped skunk rabies remains largely if not fully unmanaged, and there are no efforts undertaken in the region to actively monitor disease prevalence. An important cause for concern as a result of this is the potential for skunk variant virus to spillover to non-reservoir species, including domestic animals, which occur time to time in the North Central Plains and other enzootic regions.

Geographically scattered skunk rabies cases are usually recorded throughout the year in the North Central Plains [2]. However, the temporal and spatial dynamics of striped skunk rabies, or in other words whether or not this disease among skunks has been increasing or decreasing over the years, and whether it is expanding or contracting in geographic extent within the areas where it is known to occur is not clearly known since active surveillance is lacking. Striped skunk habitats overlap with those of humans, particularly in the periurban areas, and evidence suggests that striped skunk abundance is relatively higher in urban and forest edge environments. The magnitude of risk posed by striped skunks to humans and domestic animals in such environments are however not clear. Further, research on environmental factors, including climate and land cover that could be influencing the epizootiology of striped skunks in the north central US are not available in the literature. Wildlife mammalian distribution and their movement, and therefore the pathogens they vector are influenced by environmental and climatological pressures, some of which are undergoing rapid changes, and an understanding of such factors is crucial for effective disease management.

Our objectives in this study were to explore the spatial and temporal patterns of rabies infection status among striped skunk cases submitted for testing in the North Central Plains, and to evaluate any influential environmental and climatological factors that drive such patterns. We hypothesized that the spatio-temporal patterns of passively surveilled striped skunk rabies cases can be described by modeling the underlying spatially structured and unstructured heterogeneities, nonparametric time trend and space-time interaction in a Bayesian hierarchical construct; and, that such a model can be extended to identify important eco-climatic drivers of such patterns, whose posterior estimates when further exponentiated can be used to describe the risk of skunk to human rabies transmission and as well as the risk of enzootic skunk rabies.

## Materials and Methods

Rabies infection data on striped skunks for the region are rarely collected using active surveillance methods. We therefore used a passive surveillance data maintained at the Kansas State Veterinary Diagnostic Laboratory (KSVDL), and for the eco-climatic variables utilized publicly available data repositories (NASA, USGS, and US Census Bureau). The geospatial analyses were performed in ArcGIS environment and models were constructed using a Bayesian hierarchical framework in R-INLA (Integrated nested Laplace approximations). The following sections present specific details.

### Striped skunk rabies data

This study utilized a retrospective case-control study design, with samples for cases and controls obtained from medical records maintained at KSVDL. Records of tests performed for the presence of rabies virus in striped skunk tissue samples that were submitted during January 2007 –December 2013 were obtained from a laboratory information management system. Observations were coded in a binary fashion, those with positive test result (case) as ‘1’, and ‘0’ for negative test result (control). KSVDL receives test requests for rabies predominantly from the US states of Kansas and Nebraska, and records from these states alone were kept for statistical evaluations. Address information provided along with submission forms were geocoded in ArcGIS environment for mapping and were projected into North American Datum of 1983 (NAD83) State Plane coordinate system. Presence of address locations within rural *vs*. urban areas was assessed in ArcGIS using US Census Bureau’s 2010 census urban and rural classification criteria. Urban areas according to US Census Bureau’s definition includes densely developed territory and encompass residential, commercial and other non-residential urban land uses. The US Census Bureau identifies two types of urban areas, *viz*., urbanized areas of 50,000 or more people and urban clusters of at least 2,500 but less than 50,000 people. Any other territory, population or housing not included in ‘urban’ are considered rural [12].

### Eco-climatic covariates

Striped skunk home range is sex and age dependent and could vary from 3.75–5.0 km^2^ [13–15]. We assumed an area covering 4 km^2^ as home-range for the purposes of this study. A polygon layer with 4 km^2^ area was created in ArcGIS, and environmental and climatic data were extracted from publicly available sources surrounding case locations. The 2006 National Land Cover Dataset [16] was obtained from the United States Geological Survey (USGS) in a raster grid format. Grids representing different land cover type within each areal unit (4 km^2^ area) were extracted from the raster dataset and the percentage area they occupy were estimated. A list of land cover variables evaluated in the study is present in Table 1. In addition to deriving percent land-cover areas, a landscape metric, Total Edge Contrast Index (TECI) was derived. TECI was calculated in FRAGSTATS [17] program by

$$TECI={\left[\sum_{i=1}^{m}\sum_{k=i+1}^{m}e_{ik}d_{ik}\right]}^{-E^{*}}(100).$$

where *e*_*ik*_ is the total length of edge between patch types *i* and *k*, and E*is the total length of edge in landscape, and *d*_*ik*_ is the dissimilarity (edge contrast weight) between patches *i* and *k*.

**Table 1:** Results of univariate logistic regression analysis (frequentist) of candidate covariates evaluated in the study (*P<*0.2).

Source/variable	Control (Mean ± S.E)	Case (Mean ± S.E.)	*p* ≤ 0.2
***National Land Cover Dataset***			
*a*. *Percentage ground cover*			
Open water	0.83 ± 0.18	0.75 ± 0.11	0.65
Developed—open space	1.54 ± 0.54	1.08 ± 0.81	0.41
Developed—low intensity	1.41 ± 1.01	3.02 ± 0.77	0.02
Developed—high intensity	2.44 ± 1.63	2.11 ± 2.16	0.42
Barren land	1.81 ± 0.68	2.21 ± 1.01	0.54
Deciduous forest	8.41 ± 2.74	9.27 ± 2.33	1.48
Mixed forest	2.14 ± 1.20	2.98 ± 0.58	0.29
Evergreen forest	10.24 ± 3.54	8.25 ± 2.89	0.61
Scrub/shrub	7.24 ± 2.39	6.74 ± 3.04	0.74
Grassland/herbaceous cover	0.84 ± 0.01	2.11 ± 0.20	0.09
Pasture/hay	12.36 ± 5.63	10.51 ± 5.21	0.35
Woody wetlands	1.36 ± 0.85	2.11 ± 1.02	0.71
Emergent herbaceous wetlands	1.55 ± 1.02	1.34 ± 0.87	1.88
*b*. *Landscape metric*			
Total edge contrast index (TECI)	1.84 ± 0.21	2.81 ± 0.42	0.03
*NASA Moderate Resolution Imaging Spectrometer (MODIS)*			
*a*. *Daytime land surface temperature*			
≥35°C	36.32 ± 3.14	38.21 ± 2.36	0.61
28–34.9°C	30.95 ± 1.25	29.22 ± 1.23	1.10
24.9–27.9°C	26.54 ± 1.02	26.11 ± 0.84	1.51
≤25°C	Reference category		
*b*. *Night time land surface temperature*			
≤16°C	Reference category		
15.9–19.9°C	16.44 ± 1.21	16.14 ± 1.25	1.21
≥20°C	21.33 ± 0.88	23.01 ± 1.05	0.19
*c*. *Diurnal temperature range*	12.00 ± 0.14	15.21 ± 0.41	0.04
*NASA Prediction of Worldwide Renewable Resources*			
Daily maximum temperature	34.32 ± 1.22	34.62 ± 1.24	1.84
Daily minimum temperature	15.25 ± 1.55	16.05 ± 1.50	0.57
Daily average temperature	28.44 ± 2.61	27.58 ± 2.07	0.66
Dew point	63.32 ± 10.21	59.21 ± 7.32	0.75
Relative humidity	74.21 ± 8.21	75.38 ± 6.32	0.81
Diurnal temperature range	11.14 ± 1.51	13.21 ± 1.32	0.06

A total of 23 variables were considered for univariate evaluations. There were 14 variables derived from the NLCD, 3 variables from NASA’s MODIS and 6 from POWER sources. All variables except daytime land surface temperature and night-time land surface temperature were in continuous form. ≤25°*C* and ≤16°*C* were used as reference categories in the models for daytime land surface temperature and night-time land surface temperature, respectively. Six variables retained significance in the univariate screening, with a liberal *p*−*value* ≤ 0.2. They were, developed—low intensity (*p* = 0.021), grassland/herbaceous cover (*p* = 0.092), grassland/herbaceous cover (*p* = 0.122), total edge contrast index (*p* = 0.033), night time land surface temperature (≥20°*C*) (*p* = 0.191), and diurnal temperature range (DTR) (*p* = 0.044).

Climatic variables including the maximum normalized vegetation index (NDVI), minimum land surface temperature *LST*_min_, mean *LST*_(min)_, diurnal temperature range (DTR) (the difference between daily maximum and minimum temperatures averaged over a thirty day period), precipitation and humidity were extracted for each 4 km^2^ area in the study area. The *LST* and NDVI estimates were derived from MODIS (Moderate Resolution Imaging Spectroradiometer) imagery [18]. DTR, precipitation and relative humidity were derived from the Prediction of Worldwide Renewable Energy (POWER) web portal of the NASA Langley Research Center [19] [20]. These datasets were resampled in ArcGIS environment whenever required to derive 4km^2^ resolution data.

### Statistical analysis

#### Univariate parameter estimation

Candidate explanatory variables (Table 1) to be included in the Bayesian hierarchical models were screened *a priori* in order to avoid model fitting issues. Several frequentist bivariate logistic regression models were used to evaluate each variable independently, and only variables that were significant at a liberal *p*<0.2 were kept for further evaluation [21]. A logistic regression takes the form,

$$\text{log}\left[\pi_{ij}\right]=\beta_{0}+\beta_{ij}vk_{ij}.$$

Where *π*_*ij*_ is the probability of positive infection status in an individual striped skunk at location *i*, in year *j*, *β*_0_ the intercept coefficient, and *β*_*k*_ the coefficient for the explanatory variable *vk*_*ij*_(*k* = 1,..,*n*). Care was taken not to remove candidate variables that were deemed clinically relevant [21]. Multicollinearity among screened variables was tested by estimating the variance inflation factor (*VIF*) and all variables with a *VIF* ≥ 10 were considered to indicate multicollinearity [22], in which case, one of the variables was dropped at a time until multicollinearity was absent. Non-linearity among independent variables was evaluated at the screening stage with logistic regressions. Significant variables with non-linearity were categorized using cut-offs based on scatterplots and reevaluated in bivariate logistic regression models.

#### Model specification

For the Bayesian hierarchical data model we assumed that the rabies infection status *Y*_*ij*_ of an individual striped skunk in location *i* in year *j* followed a Bernoulli distribution with a function *π*_*ij*_ being the probability of infection. The parameter of interest *π*_*ij*_ = *p*(*y*_*i*_ = 1|*x*_*i*_), where *x*_*ij*_ = (*x*_*ij*1_,…,*x*_*im)*_ is the vector of *m* predictors for the *i*^*th*^ individual. For the process models, we used a logit link function in an extended generalized linear model (GLM) structure that incorporated stochastic spatial and temporal functions and as well as different environmental covariate effects. The link function (logit) was defined as $\text{logit}\left(\pi_{ij}=\text{log}\left[\frac{\pi_{ij}}{1-\pi_{ij}}\right]=x_{i}\beta \right)$ so that $\pi_{i}={\text{logit}}^{-1}(x_{ij}\beta )=\frac{\text{exp}(x_{i}\beta )}{1+\text{exp}(x_{i}\beta )}.$ Several models that allowed us to evaluate random and covariate effects were fitted individually. First, a partial model with random effect terms for spatial and temporal processes in the data were fitted, notated as following,

$$Log(\pi_{ij})=\beta_{0}+u_{i}+v_{i}+\gamma_{j}.$$

Where, *β*_0_ (intercept) represent positive striped skunk rabies infection in all locations in all years, and *u*_*i*_ and *v*_*i*_ are random terms accounting for spatially structured variation in striped skunk rabies infection and unstructured heterogeneity in the data, respectively. No interaction effect was assumed to exist between *u*_*i*_ and *v*_*i*_, and these terms were assigned *u*_*i*_ ~ *CAR*, and $v_{i}\sim Normal(0,\sigma_{v}^{2})$ priors [23] [24]. Spatial dependence in *u*_*i*_ was applied by assuming a conditional autoregressive model (*CAR*)(γ) with a Gaussian distribution, which implies that the observations for each *u*_*i*_ is conditional on the neighbor *u*_*j*_ with variance $\left(\sigma_{i}^{2}\right)$ dependent on the number of neighboring 4km^2^ home range cells *n*_*i*_ of the home range cell *i*, i.e.,

$$u_{i}|u,j\text{ neighbor of }i\sim N(\frac{1}{n_{i}}\gamma \Sigma_{j=1}^{n_{i}}u_{j},\frac{\sigma_{i}^{2}}{n_{i}}).$$

The temporal component of the data was accounted for by including γ_*j*_, non-parametric term that follows a structured temporal pattern with a random walk prior of first order (RW1)$\gamma_{j}\sim N(\gamma_{j-1},\tau_{\gamma }^{-1})$[25] [26].

In addition to these terms, in a second intercept-only model, quantification of any spatio-temporal interaction effect in the data attempted by including a *Ψ*_*ij*_ term,

$$Log(\pi_{i}{}_{j})=\beta_{0}+u_{i}+v_{i}+\gamma_{i}+\Psi_{ij}.$$

which had a *Ψ*_*ij*_ ~ (*Ψ*_*i*,*j*−1_ τ_*Ψ*_) prior [27].

For the full models that included covariate terms, different covariates were included to the intercept-only model in several steps, starting with a model that included all covariates followed by removal of one covariate term at each step. No covariates were added to the intercept-only model with the space-time interaction term, *Ψ*_*ij*_ since this addition did not improve model performance over the intercept-only model without this term. Covariates were retained in the model unless their removal resulted in the increase of DIC value by 5 units or more. Terms representing several two-way covariate interaction effects were evaluated in these steps as well.

#### Model implementation and validation

Model posterior parameters were estimated using a Bayesian framework implemented using R-INLA software [28] on a Linux Beocat cluster computing environment [29]. We used non-informative, uniform priors for the distributions of covariate effects (both regression parameters, *β*_*k*_ and their variance components,$\sigma_{k}^{2}$). This allowed the observed data to have the greatest influence on posterior distributions without being constrained by the choice of prior [30]. The median estimates from the posterior distribution were calculated and exponentiated to provide odds ratios (ORs) and their corresponding uncertainty measures.

Models were validated by randomly partitioning the predicted posterior estimates into five subsets and by running the models using only four of the five subsets, while validating model prediction with the fifth subset. The models were run five times to allow each validation with subset. Each time, the model’s performance (prediction accuracy) was measured using area under the receiver-operator’s curve (AUC) values with the observed infection rates (dichotomized as 0 or 1). The mean error and mean absolute error were calculated to quantify prediction bias and overall precision respectively.

## Results

**Fig 1:**
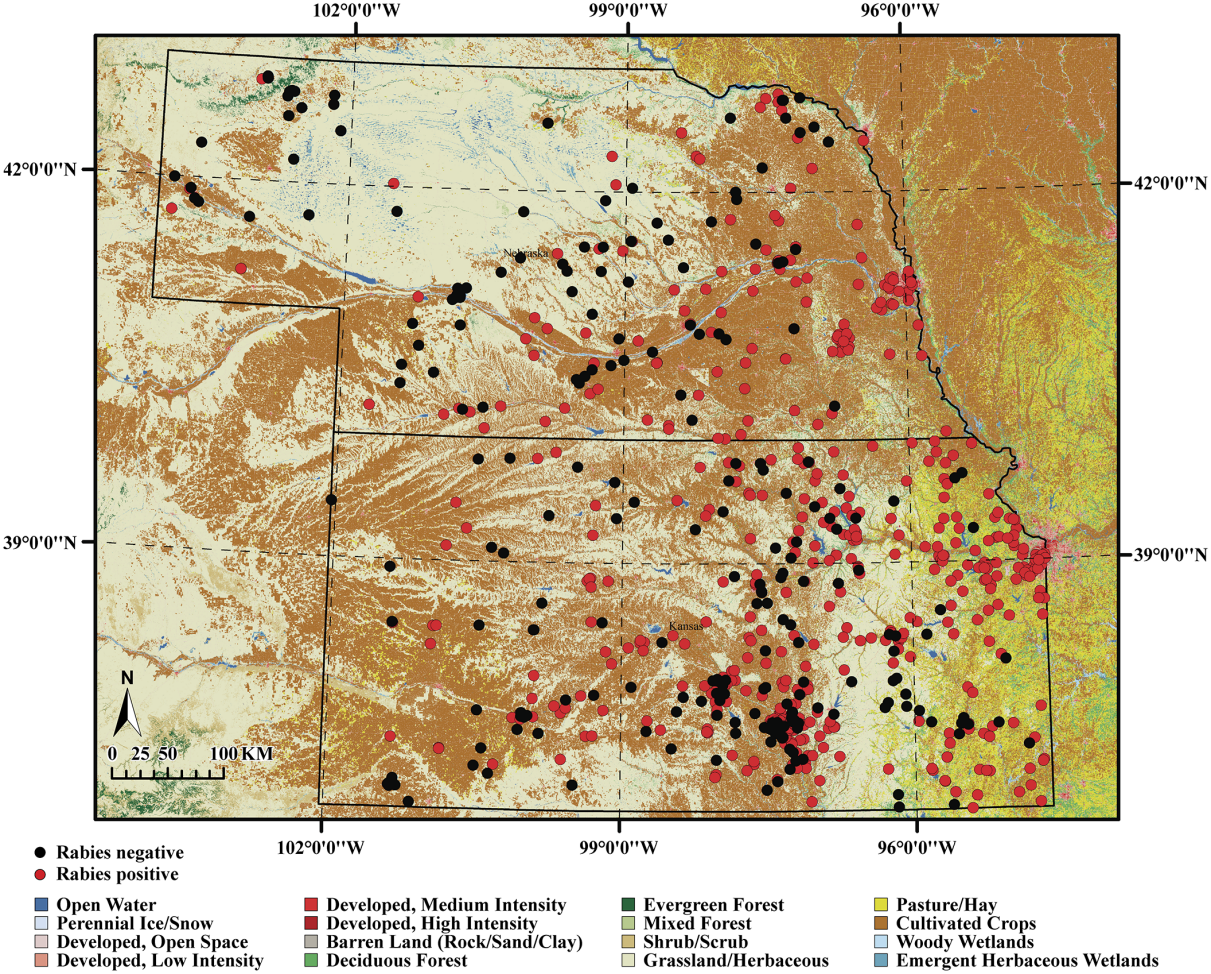
Spatial distribution of positive (dark circles) (n = 310) and negative (open circles) (n = 656) test results for striped skunk rabies in the study region.

A total of 1027 tests were performed for rabies virus on striped skunk cases in the region during the 2007–2013 study period. Among these, 705 specimens had negative diagnosis and 318 were tested to be positive. During the same period 33 specimens were determined unsuitable for diagnostic testing, and were not included in the analyses. Geographic coordinates were obtainable for 656 (93%) negative and 310 (97.4%) positive specimens, whose spatial distribution is present in Fig 1. A predominant number of striped skunk specimens tested had originated from locations that were completely present within areas classified as rural (*n =* 905, [93.7%]) and the remaining (*n =* 61, [6.3%]) within urban boundaries.

Of twenty three covariates evaluated in the univariate parameter estimation, six were retained for Bayesian hierarchical analysis (Table 1). No multicollinearity was noted among the retained covariates, and univariate non-linearity in logit with relation to positive infection among striped skunks over time and geographic extent was not noted.

**Fig 2:**
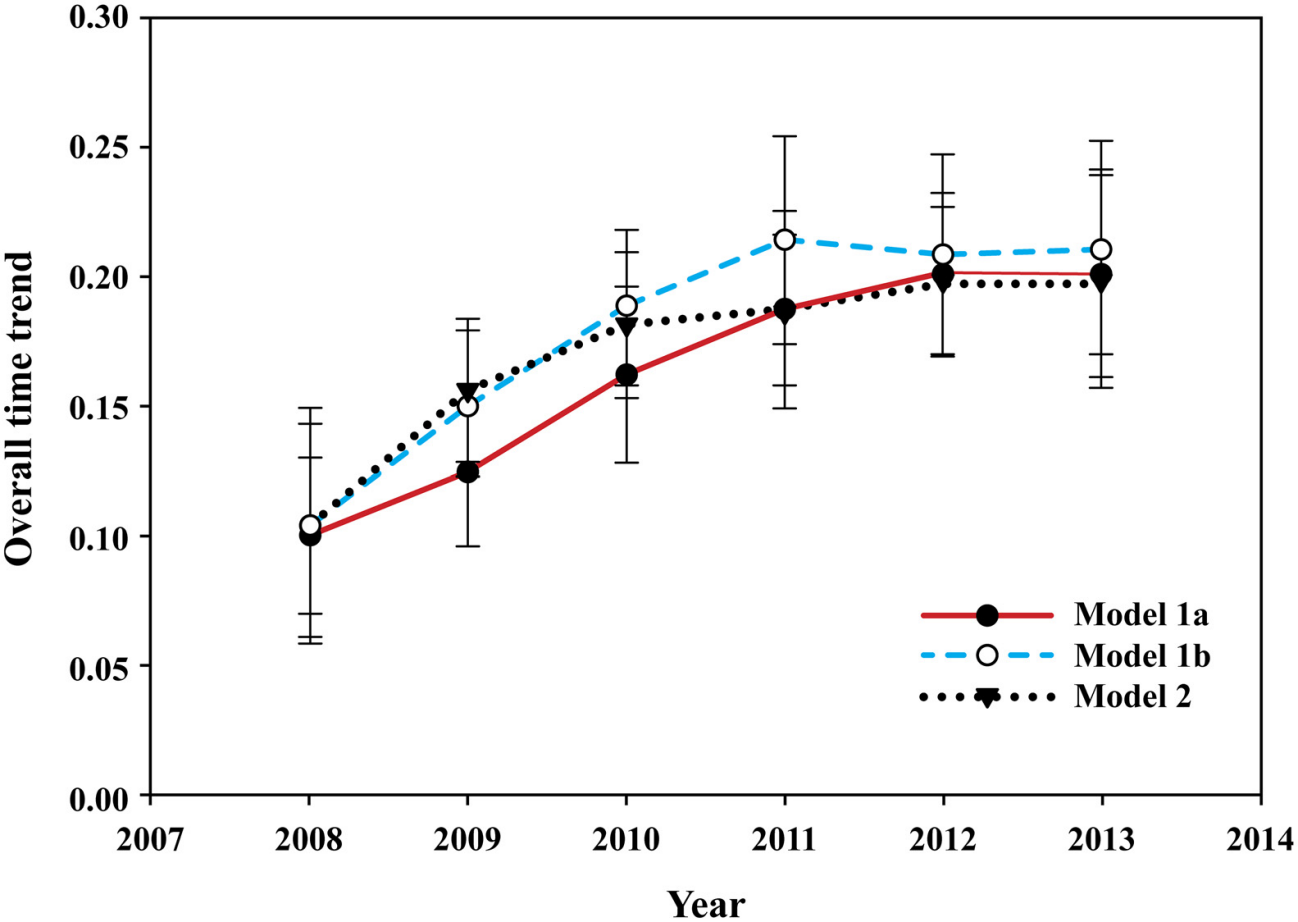
Overall time trend for the three models with the non-parametric γ_*j*_ term including 95% credible intervals.

The covariate model with independent random effects for spatial and temporal terms, and fixed land cover/land use and climatological covariates performed best among the three Bayesian hierarchical models considered in this study, indicating that the inclusion of covariate terms explained additional variability among striped skunk rabies submissions that were unaccounted by purely random-effect terms. The summary of obtained posterior estimates for the hyperparameters (*τ*_*u*_,*τ*_*v*_ and *τ*_*ψ*_) in the three models (Table 2) showed that the spatially unstructured regional effect, indicating spatial heterogeneity, and the spatially structured random effects, indicating spatial clustering were significant in all models, while the spatio-temporal effect was negligible. The estimates for the former terms decreased in the covariate model, likely due to the fixed effect covariate terms competing to explain the same processes in the model. The non-parametric term, γ_*j*_ for structured temporal trend in all three models (Fig 2) indicated that there was an increasing overall time trend, albeit by very small amounts in the data during the initial years followed by a stable trend throughout the remaining years in the study.

**Table 2:** Model statistics from two spatio-temporal models evaluating striped skunk rabies incidence in Kansas, Nebraska in the Northern Plains, USA.

Parameter	Partial model (1a)	Partial model (1b)	Covariate model
*Random effect terms* (*Mean ± SD*) ^£^			
*β*_0_	0.35 ± 0.04	0.33 ± 0.04	0.28 ± 0.03
*u*_*i*_	0.04 ± 0.00	0.06 ± 0.02	0.02 ± 0.01
*v*_*i*_	0.18 ± 0.02	0.11 ± 0.01	0.08 ± 0.02
*γ*_*j*_	0.23 ± 0.05	0.20 ± 0.05	0.21 ± 0.05
*Ψ*_*ij*_	-	-0.03 ± 0.05	-
*Fixed effect covariates (Odds ratio*, *95% Credible Intervals)*^¶^			
*β*_1_ (% developed—low intensity areas)	-	-	3.41 (2.01, 3.83)
*β*_2_ [Total edge contrast index (fragmentation)]	-	-	1.70 (1.26, 2.81)
*β*_3_ (Diurnal temperature range)	-	-	0.54 (0.27, 0.91)

^£^ Mean and standard deviation correspond to the posterior estimates for the hyperparameters *τ*_*u*_,*τ*_*v*_,*τ*_*γ*_, and *τ*_*ψ*_ in the three Bayesian models present above.

^¶^ The odds ratio and credible intervals correspond to the median of the posterior predictive distributions of the covariate model.

*β*_0_ is intercept in all models, representing positive striped skunk rabies infection in all locations in all years, and *u*_*i*_ and are *v*_*i*_ random terms accounting for spatially structured variation in striped skunk rabies infection and unstructured heterogeneity in the data, respectively. *γ*_*j*_ and *Ψ*_*ij*_ terms represent non-parametric time trend and spatio-temporal interactions, respectively. Information on the choice of priors for these terms are provided in the text.

The covariate model indicated that higher percentages of low intensity—developed areas, total edge contrast index (henceforth referred to as patch fragmentation) within skunk home ranges (4 km^2^ units), and a climate variable, diurnal temperature range were significantly associated with positive rabies infection among submitted cases of striped skunks (Table 2). All further interpretations are made on this model alone. The ORs and 95% Bayes CrIs indicate the risk of rabies infection among striped skunks submitted for testing in the study region for each of the 4 km^2^ habitat cells. For every percentage increase in developed—low density area within a 4 km^2^ habitat cell, the odds of a striped skunk specimen collected within that cell to test positive for rabies is 3.41 times larger than being negative. Likewise, for every percentage increase in patch fragmentation (as measured by total edge contrast index), the odds of testing positive was 1.7 times larger than testing negative; and, for every unit increase in DTR, the odds of testing positive reduced by 0.54 times; or in other words, the ongoing decrease in DTR due to climate change increased the odds of rabies infection. Developed—low intensity areas and patch fragmentation indirectly point to the relatively higher human density in such areas, which increases striped skunk encounter rates and the probability of positive diagnosis.

The addition of an interactive space-time term, *Ψ*_*ij*_ to the partial ST model yielded a Deviance Information Criterion (DIC) value of 1,459, marginally higher than the DIC value (1,431) without the term included. Additionally, the inclusion of *Ψ*_*ij*_ term and covariate terms to the final covariate model failed to improve model performance, indicating a lack of space-time process in the dataset (Table 3).

**Table 3:** Model fit and comparison statistics.

Model	$\bar{D}$	*p*_*D*_	*DIC*
*Partial*			
(1a)	1320	81	1401
(1b)	1345	114	1459
*Covariate*^£^			
(2)	952	32	984

$\bar{D}$ = posterior mean deviance, calculated as $\bar{D}=Ε\left[D\right].$ where *D* = −2log *p(y*|*θ*).

*p*_*D*_ = Posterior mean deviance—deviance of posterior means, calculated as *p*_*D*_ = *E*_*θ*|*y*_ [*D*]−*D*(*E*_*θ*|*y*_[*θ*]).

*DIC* **=** Deviance information criterion, analogous to the frequentist AIC estimate and estimated as $DIC\;=D\left(\bar{\theta }\right)+2p_{D}.$

^£^Several covariate models (which also included random effect terms) were fitted starting with a model that included all covariates that were screened in the univariate procedure with a liberal *p* ≤ 0.2, followed by the removal of one covariate at a time from the Bayesian hierarchical models. The removal of % grassland area, minimum land surface temperature and an interaction term, ‘diurnal temperature range x % mixed forest area’ one at a time, in that order resulted in models with *DIC* values of 1261, 1014, and 1008. To the final covariate model, a random effect space-time term, *Ψ*_*ij*_ was inserted, which resulted in a *DIC* value of 1023, indicating poor performance. Other previously removed covariates did not re-enter the final covariate model.

The posterior estimates used to derive ORs and 95% CrI correspond to the median of the posterior predictive distribution of the covariate model (Table 3). Model validation statistics based on the mean error and mean absolute error derived by randomly partitioning incidence data within 4 km^2^ areas into test and model groups did not indicate model inadequacies and the area under curve (AUC) values for the covariate model indicated good discriminative capacity (Table 4).

**Table 4:** Validation statistics for the partial and covariate Bayesian models.

Model	AUC*	Mean error^†^	Mean absolute error^‡^
Partial (1a)	0.71	0.21	6.23
Partial (1b)	0.68	0.42	6.86
Covariate model	0.78	0.18	4.27

* AUC values in the range of 0.5–0.7 indicates poor discriminative capacity, 0.7–0.9 is considered good and > 0.9 to be very good.

^†^ Overall tendency to over or under-predict relative risk.

^‡^ Overall precision of models estimated using magnitude of error in predictions.

## Discussion

Understanding the spatial and temporal patterns of diseases is important for disease management. While methods have been developed to model these aspects of infectious diseases in human populations and to some extent also for companion and food animals [25] [31] [32], it is rather difficult to quantify the space-time epidemic processes for those diseases vectored by wildlife [33]. Wildlife diseases are not regularly monitored, and even at times when they are studied through well thought-out sampling regimens the infection rates can be heterogeneous over different landscapes in addition to being confounded by host factors such as age, sex and genotype [34] [35]. This calls for the analysis of such infection data using statistical methods that are well equipped to account for spatial, temporal and host-level heterogeneities. Another issue, particularly with striped skunk rabies but likely also with other rare wildlife diseases is the general lack of active surveillance data. Sampling efforts to quantify rabies prevalence among striped skunks in the region are not found even though evidence for rabies in this host in the Northern Plains region has been known for many decades [5] [6]. However, a different source for disease prevalence information are diagnostic laboratories where testing for diseases is routinely performed. The Rabies Laboratory at KSVDL is one of the largest diagnostic facilities in North America, and the primary diagnostic lab for the states of Kansas and Nebraska where skunk rabies is a reportable disease. We used retrospective disease information gathered at this facility, and for the statistical analyses employed Bayesian hierarchical models in order to alleviate the difficulties in modeling uncertainties associated with retrospective data with an inherent spatio-temporal structure [31] [36].

The use of passive surveillance data such as this introduces two important limitations, due to which the findings reported here are only directly applicable to a sub-population of skunks. It is likely that many of the skunks submitted for testing were those that had human/domestic animal encounter or those that showed visible signs of malady to passing-by humans, whereby introducing a population bias. And, it is also quite likely that these skunk samples originated from areas closer to human dwellings and not from a wider habitat, and therefore introducing a spatial bias. Even though a spatial analysis of sample locations in this study revealed that a predominant number of cases originated from rural locations (93.7%) with human population less than 2500 persons, and which have land cover characteristics akin to those of wild skunk habitats (except for any heavily developed areas), a distinction must be made in the interpretation of the results of this study to a sub-population of striped skunks and not the entire striped skunk population. Carefully designed prospective studies will be needed to fully alleviate all population and spatial biases. The present study underscores a need for such an effort since any and all of the information currently available on the space-time dynamics or eco-epidemiological drivers of striped skunk rabies exist only in the form of passive surveillance data.

Of all the Bayesian hierarchical models that were evaluated in the study, the covariate model that included independent random terms for spatial and temporal effects and fixed eco-climatological covariates had the highest potential in explaining rabies incidence in striped skunks. The covariate model included non-interactive spatial and temporal terms and they significantly accounted for some of the variability in the incidence data. This indicated that the odds of positive infection presence in a given year in any one of the 4 km^2^ home range is dependent on the infection presence in the neighborhood in the previous year or years. Despite this finding, we did not note any significance for the interactive spatio-temporal term in the partial or covariate models. Unlike non-interactive spatial and temporal terms, the presence of a significant space-time interaction in a dataset is often an indication of the presence of localized clusters that may be linked, for instance to emerging ecological pressures to hosts. Our finding of a lack of significant space-time interaction does not however mean that rabies among submitted striped skunk cases has not spread across the landscape or that the prevalence has remained constant within the study region. In fact, the clumped case distribution (Fig 1) in some areas but not others suggests that submissions of striped skunk cases for rabies testing has expanded to larger area in the past, perhaps at a slower rate, and the measurement of spatially explicit temporal changes are undetectable over moderate time periods and smaller spatial scales despite the use of sophisticated methods. Again, carefully designed prospective studies are warranted that would help assess the dynamics of striped skunk rabies prevalence and/or its spread and intensity in the region.

The covariate model in the present study had an AUC value of 0.78 (Table 3), which indicated a reliable model performance, reflecting that a linear combination of fixed covariate and random effect terms predicted the infection prevalence in the 4 km^2^ home range locations with satisfactory level of accuracy. This model has also identified important eco-climatological drivers for striped skunk rabies in the region based on passive surveillance, which to the best of our knowledge are not available in the published literature. The ORs and 95% Bayes CrIs associated with the covariates indicate the statistical odds of detecting positive rabies infection among striped skunk cases submitted for testing and their corresponding uncertainty levels. In epidemiological studies the ORs are interpreted in terms of infection or disease risk. Three covariates were retained in the model; developed—low intensity areas, landscape fragmentation, and diurnal temperature range (DTR), and they simultaneously indicate risk of rabies infection from striped skunks to humans and as well as the enzootic risk among striped skunks. The higher percentages of developed—low intensity areas and higher fragmentation on a landscape indicate the elevated risk of rabies transmission to humans from striped skunks, while the decreasing DTR points to the enzootic striped skunk rabies risk in the North Central Plains.

Developed, low intensity area on a landscape is defined by USGS as areas with a mixture of constructed materials and vegetation where impervious surfaces account for 20% to 49% percent of total ground cover. These areas most commonly include single-family housing units [37], and are also likely to be newly urbanized portions of cities with potentially high human-wildlife interactions [38]. It is well documented that exurbanization, or the increasing tendency for urban residents to settle in suburban or rural environments increases anthropogenic influences on the landscape and concentrate vital resources for wildlife [39]. Striped skunks are tolerant towards humans and when opportunities are present they are known to inhabit less accessed areas in and around human dwellings. The observed higher odds of detecting positive infection among striped skunks from such places therefore presents an elevated risk for rabies transmission to humans and as well as domestic animals in this environment.

The 4 km^2^ habitat cells in the study region with higher patch fragmentation positively impacted infection among striped skunks. At the landscape level, total edge contrast index is a measure of patch fragmentation—higher values indicate higher fragmentation. Some effects of landscape fragmentation are that it leads to more and smaller habitat patches, decreased complexity of patch shape, and higher proportions of edge habitats [40], and as a result it has been hypothesized that habitat fragmentation could lead to rise in infectious disease among wildlife by facilitating an increase in susceptible host abundance and as well as by increasing the disease load due to diminished host diversity [41] [42]. In addition, it is known that striped skunks are adapted to living in a variety of land cover types including rural and urban environments [43]. Unlike other wildlife mammals such as opossums and raccoons, striped skunks generally do not show a preference to any one particular land use type [44] [45]; however, previous studies have shown that their abundance was highest near urban edges [46] [47] and forest edges [48], and skunk dispersal is increased along edge habitats between fields and forest fragments [49]. For these reasons, it is plausible that higher skunk population in fragmented areas lead to more submissions resulting in a higher detection rate, without a true increase in rabies incidence rate. Regardless of the process involved, we suspect that highly fragmented landscapes in the study region at the edges of urban development and around agricultural fields pose a significant risk of rabies infection to humans and domestic animals since they favor striped skunk habitats.

Climate plays an important role in striped skunk life history and foraging behavior. The spatio-temporal changes in temperature, precipitation and humidity that are expected to occur under different climate-change scenarios will affect the biology and ecology of disease vectors, intermediate hosts and consequently the risk of disease transmission [50]. The identification of diurnal temperature range as a driver for striped skunk rabies in the region is therefore significant, since it has been identified as an index of climate-change [51] [52], and further field experiments to quantify this linkage could help us understand climate-change impacts on wildlife disease ecology, including striped skunk rabies. DTR has narrowed over the US since the 1950s at least in part due to differential changes in daily maximum and minimum temperatures [53]. For most parts of the US, trends show that *T*_*max*_ have remained constant or have increased only slightly but *T*_*min*_ have increased at a faster rate [50] [54]. There is no direct evidence in the literature so far that indicates increasing night-time temperatures (*T*_*min*_) translate to changes in rabies ecology among striped skunks, for instance *via* an extended movement period from their dens for foraging activity or other adaptations. However, during the warm winter of 2008 in Kansas striped skunks were noticed to move farther (948 ± 207 m) between locations than a typical average distance of 197 ± 44 m covered during other winters [55], and such occurrence may become more commonplace as DTR continues to change. Identifying associations between climatic factors and disease outcomes is often challenging due to other confounding factors [56] but such knowledge is vital for quantifying any role that climate-change may be playing towards the amplification and/or spatial expansion of disease incidences. Studies considering long term surveillance of striped skunk rabies prevalence in the field and its association with climate-change indices are therefore needed.

### Conclusions

This study presents current spatiotemporal pattern of striped skunk rabies in the North Central US based on passive surveillance, and identifies influential eco-climatological drivers of this disease using Bayesian hierarchical modeling approach. Developed, low intensity areas and highly fragmented landscapes are mostly periurban environments and areas where human habitats overlap striped skunk habitats. In these environments higher rates of human—striped skunk contact is possible. There is a relatively higher risk of rabies transmission from striped skunks to humans who reside in developed low intensity areas and highly fragmented landscapes such as edges of woodlands and agricultural lands than other places. Diurnal temperature range, a climate change indicator is decreasing at a slow but steady rate, and increases the enzootic risk of rabies to striped skunks. Human mediated landscape changes and climate-change appears likely to exacerbate the prevalence of this disease in this species, and further studies are necessary to more fully understand the dynamics of skunk rabies in the study region and its impact upon the prevention of rabies among humans and other animals.
